# A MYC-LDHA-MCT4 expression program marks an SPP1/TREM2-centered macrophage state in pancreatic ductal adenocarcinoma

**DOI:** 10.3389/fimmu.2026.1978427

**Published:** 2026-10-05

**Authors:** Qianqian Ma, Hequn He, Ting Liu, Ying Li, Chenyang Mao, Chao Shi, Qi Yao

**Affiliations:** 1 Department of Geriatrics, The First Affiliated Hospital of Ningbo University, Ningbo, Zhejiang, China; 2 Department of Emergency Medicine, The First Affiliated Hospital of Ningbo University, Ningbo, Zhejiang, China

**Keywords:** immunometabolism, lactate, MCT4, MYC, pancreatic ductal adenocarcinoma, SLC16A3, spatial transcriptomics, SPP1

## Abstract

**Introduction:**

Pancreatic ductal adenocarcinoma (PDAC) responds poorly to immune-checkpoint blockade, and the metabolic organization of its myeloid barrier is incompletely understood.

**Methods:**

We integrated imaging mass cytometry from the phase II trial NCT04177810, three single-cell RNA-seq datasets, two spatial transcriptomic cohorts (12 sections), three survival cohorts, and *in vitro* siRNA perturbation of MYC, LDHA and SLC16A3 with conditioned-medium assays on THP-1-derived macrophage-like cells and primary human CD8+ T cells.

**Results:**

CD8+ T-cell infiltration increased without objective responses while macrophages remained abundant. Patient-level epithelial MYC and glycolysis programs correlated with SLC16A3 (MCT4) and a macrophage lactate-reception program. Across 12 spatial sections, macrophage-receiver scores were higher around MCT4-high cores (ring 1: 12/12 positive; median difference 0.433; FDR = 0.010), with the strongest state-resolved signal for SPP1/TREM2 macrophages (median difference 0.613; FDR = 0.006); the composition-adjusted effect remained positive (10/12) but was non-significant (FDR = 0.071). Silencing MYC, LDHA or SLC16A3 reduced extracellular lactate and altered conditioned-medium effects, with partial rescue by lactate add-back. Survival associations were cohort-dependent with substantial heterogeneity.

**Discussion:**

These results define an exploratory, expression-based spatial association between a tumor MYC-LDHA-MCT4 program and an SPP1/TREM2-centered macrophage state, constituting a testable hypothesis rather than evidence of lactate transfer.

## 1 Introduction

Pancreatic ductal adenocarcinoma (PDAC) remains among the most lethal solid tumors and is characterized by early dissemination, dense desmoplasia and profound resistance to immunotherapy (1–3). Although blockade of PD-1 or CTLA-4 can produce durable responses in selected cancers, unselected PDAC generally remains non-responsive. Its immune microenvironment is dominated by fibroblastic and myeloid programs that restrict productive T-cell entry and function (4–7). A recent phase II trial combining CXCR4 antagonism with anti-PD-1 therapy illustrated this paradox: treatment recruited T cells into PDAC lesions but failed to generate objective responses and was accompanied by immunosuppressive macrophages (8). This clinical observation suggests that immune recruitment alone is insufficient when a metabolically adapted myeloid barrier persists.

Metabolic reprogramming is a plausible organizer of this barrier. MYC coordinates glucose uptake and glycolysis, LDHA converts pyruvate to lactate, and the proton-linked transporter MCT4, encoded by SLC16A3, exports lactate from highly glycolytic cells (9–12). Extracellular lactate and acidosis suppress cytotoxic lymphocytes, alter dendritic-cell function, and promote macrophage states associated with angiogenesis, matrix remodeling and immune regulation (13–17). Lactate can additionally serve as a carbon source and signaling metabolite, and lactylation provides a route through which metabolic state can be recorded in chromatin (18, 19). Nevertheless, transcript abundance is not equivalent to lactate flux, and most bulk-tumor studies cannot establish which cells generate or receive a metabolic signal or where those cells are positioned. The recurrent PDAC driver alterations KRAS, TP53, CDKN2A and SMAD4 provide a genomic background for these phenotypes. In particular, oncogenic KRAS can increase glucose uptake and anabolic glucose metabolism, but the present datasets were not designed to test mutation-specific regulation of the expression program (20–22).

SPP1- and TREM2-expressing macrophage states have been described in PDAC and other treatment-resistant tumors, with functions that vary by tissue and disease context (23, 24). Yang et al. examined the inflammatory consequences of TREM2 perturbation in pancreatic cancer, whereas the present study asks whether an SPP1/TREM2-centered macrophage state is spatially positioned near an epithelial MYC/LDHA/SLC16A3 expression program. Tang et al. (25) provided cell-type-resolved metabolic characterization of the PDAC microenvironment; our analysis adds a section-level donor-receiver spatial hypothesis across two independent Visium cohorts. Gao et al. integrated single-cell, spatial and GSE62452 data to characterize lactate metabolism in PDAC broadly (26). Our incremental contribution is therefore limited to the convergence of the MYC/MCT4 tumor axis, an SPP1/TREM2-centered receiver state, cross-cohort spatial colocalization, conditioned-medium perturbation, and a translational entry point from the NCT04177810 imaging-mass-cytometry dataset. We do not claim novelty for the general relationship between lactate metabolism and PDAC immunity.

## 2 Materials and methods

### 2.1 Study design and public datasets

The study combined tumor-cell experiments with secondary analysis of de-identified public datasets. Imaging mass cytometry from the phase II metastatic PDAC trial NCT04177810 of CXCR4 inhibition plus anti-PD-1 therapy was used to define the clinical problem of immune recruitment without objective response (21 treated patients; 11 with paired evaluable tissue) (8). Single-cell analyses used GSE154778 (24) and GSE155698; tumor-versus-blood myeloid validation used GSE217845 (27). Spatial analyses used five treatment-naïve PDAC sections from GSE274103 (28) and seven independent sections from GSE235315 (29). Survival-cohort eligibility required PDAC tissue expression data, individual overall-survival time and status, and measurement of all nine score genes on the same platform. TCGA-PAAD, GSE62452 and GSE71729 met these criteria and were retained regardless of effect direction. TCGA-PAAD was designated the discovery cohort; both GEO cohorts were external sensitivity cohorts. Dataset accessions, sample flow, event numbers and analysis roles are reported in **Supplementary Table 1**.

### 2.2 Single-cell processing and annotation

Sparse count matrices were imported into Seurat 5.5.1. For GSE154778, cells were retained when 200-7,500 genes were detected, mitochondrial reads were <=20%, and total UMI counts were below the patient-specific 99.5th percentile. Doublets were called within patient using scDblFinder 1.24.0 (30) with SerialParam and removed. For GSE155698 and GSE217845, cells with 300-7,500 detected genes and <25% mitochondrial reads were retained; the available processed matrices did not support reconstruction of every prefiltering intermediate. Counts layers were joined before conversion to SingleCellExperiment. Data were log-normalized; 3,000 variable genes were used where specified. For GSE154778, PCA used 40 components, Harmony 1.2.3 (31) was run by patient on dimensions 1-40, neighbors and UMAP used Harmony dimensions 1-30, and clustering was evaluated at resolutions 0.4, 0.6 and 0.8, with 0.6 used for downstream labels. The random seed was 20260723. No cell-cycle regression was applied. Broad lineages were assigned from canonical markers and compared with SingleR 1.16.0 (32) labels generated from the Human Primary Cell Atlas reference in celldex 1.22.0; pruned cluster labels were retained for review but marker-supported lineage calls were used in downstream analyses. Malignant epithelial candidates were defined by epithelial identity and a chromosome-window expression score constructed from autosomal gene-order bins, using low-CNV epithelial and non-epithelial populations as internal references; this is an expression-based proxy rather than DNA copy-number measurement. Macrophage states were assigned by maximum standardized module score and checked against cluster markers. Cell counts after available QC steps are reported in **Supplementary Table 1**; complete gene sets are given in **Supplementary Table 2**.

### 2.3 Expression-program scores and patient-level association tests

Module scores were calculated as the mean log-normalized expression of all available genes in each listed set (**Supplementary Table 2**). Scores used as predictors and the genes used as measured outcomes were kept separate in each inferential test; where a target gene was plotted descriptively within its own state module, no inferential claim was based on that mathematical association. Within each dataset, component scores were standardized before combination. Cell-level values were aggregated to patient and lineage before association testing. Two-sided Spearman correlations were calculated at patient level and are reported with rho, sample size and P or FDR; confidence intervals were not generated in the original pipeline and this limitation is stated. Macrophage differential expression used patient-by-state pseudobulk raw counts and edgeR (33), followed by ranked Hallmark enrichment. Dataset-specific estimates were retained rather than pooling cells across cohorts.

### 2.4 Spatial transcriptomic analysis

Visium filtered-feature matrices and tissue coordinates were processed separately for each section in Seurat 5.5.1. Only barcodes called as tissue spots by the deposited Space Ranger output and having valid coordinates, library size and component scores were eligible. The unadjusted MCT4/export core was the sum of within-section standardized epithelial, lactate-production, lactate-export and SLC16A3-expression components. High and low regions were defined statically as the upper and lower quartiles of the section-specific score; middle-quartile spots were not used as comparators. Nearest-neighbor ranks 1-6, 7–18 and 19–36 defined rings 1-3, respectively; these are spot-count neighborhoods rather than calibrated physical-distance intervals. A spot could contribute to neighborhoods of more than one qualifying core, and no explicit boundary correction or deduplication of overlapping neighborhoods was applied. Spot-level values were therefore summarized within section before inference, and the tissue section, not the spot, was the independent unit. For each section and ring, the mean value around high-score cores minus the mean value around low-score cores was calculated as a paired within-section contrast; the 12 section-level contrasts were then tested with a two-sided Wilcoxon signed-rank test and Benjamini-Hochberg correction across the defined target-by-ring family. The macrophage-receiver score combined standardized macrophage-marker and lactate-reception components. Reference mapping used Seurat FindTransferAnchors with reduction=‘pcaproject’ and dimensions 1-30, followed by TransferData with the same dimensions (34); prediction scores were retained, but no uncertainty-based spot exclusion was applied. The five GSE274103 and seven GSE235315 sections were processed separately without Harmony or ComBat integration, cohort-specific results were retained, and only section-level effect estimates were combined for cross-sample summaries. The frequency of malignant epithelial and SPP1/TREM2 macrophage signals within the same Visium spot was not estimated as a separate endpoint, limiting exclusion of density-driven colocalization. In adjusted analyses, export and SLC16A3 components were residualized for epithelial abundance and log library size, and receiver signals for myeloid abundance and log library size. Hypoxia and proliferation scores were assessed in secondary sensitivity analyses. No within-section permutation null, continuous-threshold model or formal edge correction was implemented; these omissions limit spatial inference to section-level colocalization and are not treated as evidence of transfer or causal direction.

### 2.5 Cell culture and genetic perturbation

PANC-1 and MIA PaCa-2 cells were cultured in DMEM containing 10% fetal bovine serum and 1% penicillin-streptomycin at 37 °C and 5% CO_2_. Cells were authenticated and confirmed Mycoplasma-negative. MYC, LDHA and SLC16A3 were silenced with independently validated siRNAs; a non-targeting siRNA served as control. Knockdown was assessed 48 h after transfection by quantitative reverse-transcription PCR and 72 h after transfection by immunoblotting where indicated. The original PANC-1 experiments additionally assessed CCK-8 proliferation, colony formation, Transwell migration/invasion and PI3K/AKT/mTOR-associated proteins using previously optimized protocols.

### 2.6 Lactate-output and conditioned-medium experiments

After transfection, cells were washed and placed in equal volumes of standardized medium. Conditioned medium was collected after 24 h, cleared by centrifugation and normalized to viable-cell number. L-lactate was measured with an enzymatic assay against a standard curve; glucose and pH were measured in parallel. Experimental groups comprised si-NC, si-MYC, si-LDHA, si-SLC16A3, si-SLC16A3 plus sodium L-lactate add-back to the concentration measured in control conditioned medium, and an osmolarity/pH-matched control. Cell viability was measured to exclude nonspecific toxicity. Because MYC, LDHA and SLC16A3 perturbation can alter metabolites and secreted factors beyond lactate, the conditioned-medium experiments were interpreted as supportive rather than lactate-specific. A selective MCT4 transport inhibitor and direct lactate exposure across matched pH conditions were not evaluated.

### 2.7 Macrophage and CD8 functional assays

THP-1-derived macrophage-like cells were exposed to conditioned medium collected from PANC-1 or MIA PaCa-2 cells subjected to the indicated genetic perturbations and lactate add-back conditions. Expression of SPP1, TREM2, GPNMB, APOE, SLC16A1/MCT1, LDHB, MRC1, IL1B, S100A8, and S100A9 were quantified by RT-qPCR. Intracellular lactate uptake was measured using an enzymatic assay. THP-1-derived macrophage-like cells were used as a tractable experimental model and should not be considered equivalent to primary human macrophages.

Cryopreserved primary human peripheral blood CD8+ T cells that had undergone antibody- and cytokine-mediated activation and expansion were purchased from Procell Life Science & Technology Co., Ltd. (Wuhan, China; catalog no. CP-H467B; 1 × 10^6 cells/vial). After thawing, the cells were maintained according to the manufacturer’s instructions in the recommended complete culture medium. Cell viability was assessed using trypan blue exclusion before the co-culture experiments. The commercial primary human CD8+ T cells were co-cultured with conditioned-medium-educated THP-1-derived macrophage-like cells at a CD8+ T-cell-to-macrophage ratio of 1:1 for 24h. CD8+ T-cell proliferation was evaluated using CCK8 assess. Experimental outcomes were expressed relative to the corresponding si-NC conditioned-medium group. All experimental conditions included appropriate pH and osmolarity controls.

### 2.8 Survival modeling

Target-gene expression and clinical data were harmonized for every cohort meeting the outcome-independent eligibility criteria: TCGA-PAAD, GSE62452 and GSE71729. Within cohort, each gene was standardized across patients. The tumor-export score was the equal-weight mean of MYC, LDHA and SLC16A3; the macrophage-adaptation score was the equal-weight mean of SPP1, TREM2, GPNMB, SLC16A1, LDHB and APOE; and the combined score was the standardized sum of the two standardized axis scores. Because no dated protocol, registry entry or archived pre-analysis code establishes prospective specification, all scores and survival analyses are described as exploratory. Continuous Cox models (35) per SD were primary analytical summaries; median splits were used only for visualization and were not optimized. TCGA models were adjusted for age, sex and stage, and GSE62452 for stage where available. All three eligible cohorts were included in fixed- and random-effects summaries. Leave-one-cohort-out results were interpreted descriptively because only three cohorts were available. No claim of clinical prediction is made, and calibration was not assessed. Cohort-specific sample flow, events and missing-data handling are summarized in **Supplementary Table 1**. TCGA-PAAD used RNA-sequencing data, GSE62452 used the Affymetrix Human Gene 1.0 ST platform (GPL6244), and GSE71729 used a two-channel microarray platform (GPL20769). Detailed treatment histories were not uniformly available across the public cohorts and were not used as eligibility criteria or model covariates.

### 2.9 Statistical analysis

Patient or tissue section was the independent unit. Paired IMC and tumor-versus-blood comparisons used two-sided paired Wilcoxon signed-rank tests; patient-level associations used two-sided Spearman correlation; and section-level radial differences used two-sided Wilcoxon signed-rank tests. Benjamini-Hochberg correction was applied within each stated analysis family. FDR<0.05 was considered statistically significant; FDR = 0.071 is explicitly described as non-significant. Experimental comparisons used one-way ANOVA with multiplicity-adjusted *post-hoc* tests when assumptions were met and Kruskal-Wallis tests otherwise. Individual biological observations are shown where available. Error-bar definitions, statistical units, effect measures and replicate structure are summarized in **Supplementary Table 3**. The retrospective pipeline did not include formal mixed-effects models for all nested endpoints, spatial permutation tests, Schoenfeld-residual diagnostics, nonlinear Cox terms or influential-observation analyses; survival and spatial results are therefore exploratory and require independent statistical review.

## 3 Results

### 3.1 CD8 T-cell recruitment occurs without objective response and with persistent macrophage abundance

In the CXCR4-inhibitor/anti-PD-1 trial, none of 21 treated patients achieved an objective response despite evidence of immune recruitment (**Figure 1A**). In 11 paired IMC specimens, the CD8 T-cell fraction increased in 9/11 patients (median paired change 0.0069; FDR = 0.035). Macrophage abundance also increased in 9/11 patients (median change 0.0766), although this trend did not remain significant after multiplicity correction (FDR = 0.112; **Figure 1B**). In independent paired tumor and peripheral blood samples, SPP1/TREM2 and MRC1/CD163 macrophages were higher in tumor samples from all six patients (nominal P = 0.036 for each; FDR = 0.060; **Figure 1C**). Because lactate and acidosis are established regulators of macrophage function, we next asked whether a tumor-intrinsic lactate-export program is associated with these persistent myeloid states, focusing on the SPP1/TREM2 and MRC1/CD163 populations that were enriched in tumor versus blood.

**Figure 1:**
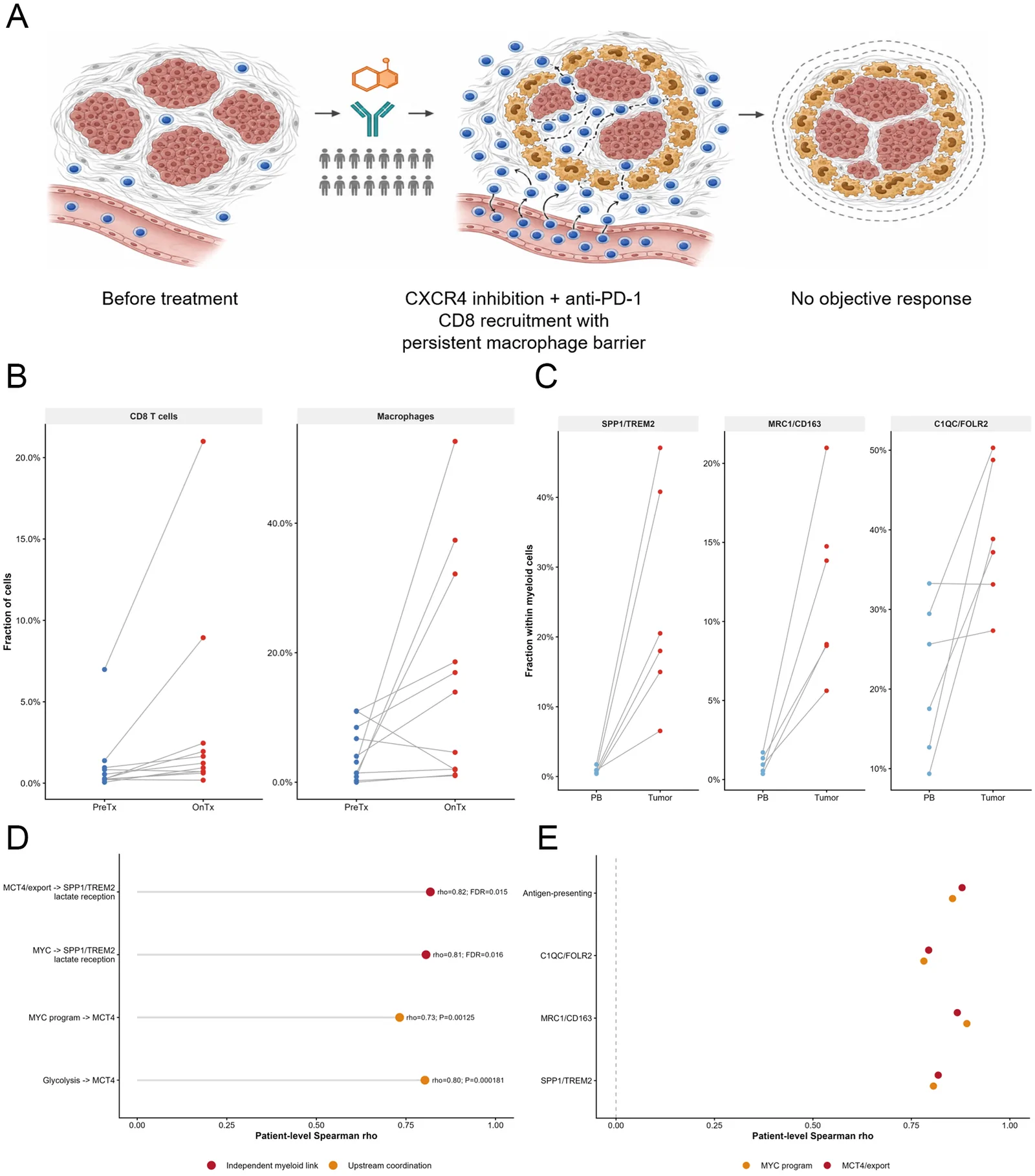
Clinical immune recruitment and cross-cohort prioritization of myeloid metabolic programs. **(A)** Clinical schematic for the phase II NCT04177810 study of the CXCR4 inhibitor motixafortide (BL-8040) with pembrolizumab-based therapy. **(B)** Paired IMC changes in CD8 T cells and macrophages. **(C)** Exploratory tumor-versus-blood macrophage-state comparison in six paired samples (n=6), raising the question of where this export program is positioned relative to macrophage-rich regions in intact tumors. **(D, E)** Patient-level associations between epithelial MYC/MCT4 programs and macrophage lactate-reception states.

### 3.2 Patient-level analyses associate MYC and glycolysis with MCT4 and macrophage receiver programs

We next examined, at the patient level, whether tumor glycolytic lactate-export programs are associated with macrophage lactate-reception programs, focusing on SPP1/TREM2 and MRC1/CD163 states, which were the two populations enriched in tumor versus blood. In GSE154778, glycolysis correlated with MCT4 (Spearman ρ=0.80, P = 1.81×10^-4^) and MYC activity correlated with MCT4 (ρ=0.73, P = 0.00125; **Figures 2B, C**). In GSE217845, epithelial MYC and MCT4/export scores correlated with the lactate-reception program of SPP1/TREM2 macrophages (ρ=0.81, FDR = 0.016 and ρ=0.82, FDR = 0.015, respectively; **Figures 1D, E**). Across two scRNA-seq cohorts, malignant candidate cells had relatively high lactate-export scores, and SPP1/TREM2 cells constituted one component of the myeloid compartment (**Figures 2D, E**). On this basis, MYC, LDHA and MCT4 were taken forward for functional and spatial analyses. On this basis, we designated the correlated epithelial glycolysis–MYC–MCT4 module as the MYC-LDHA-MCT4 lactate-export program and took it forward to functional, spatial and survival analyses.

**Figure 2:**
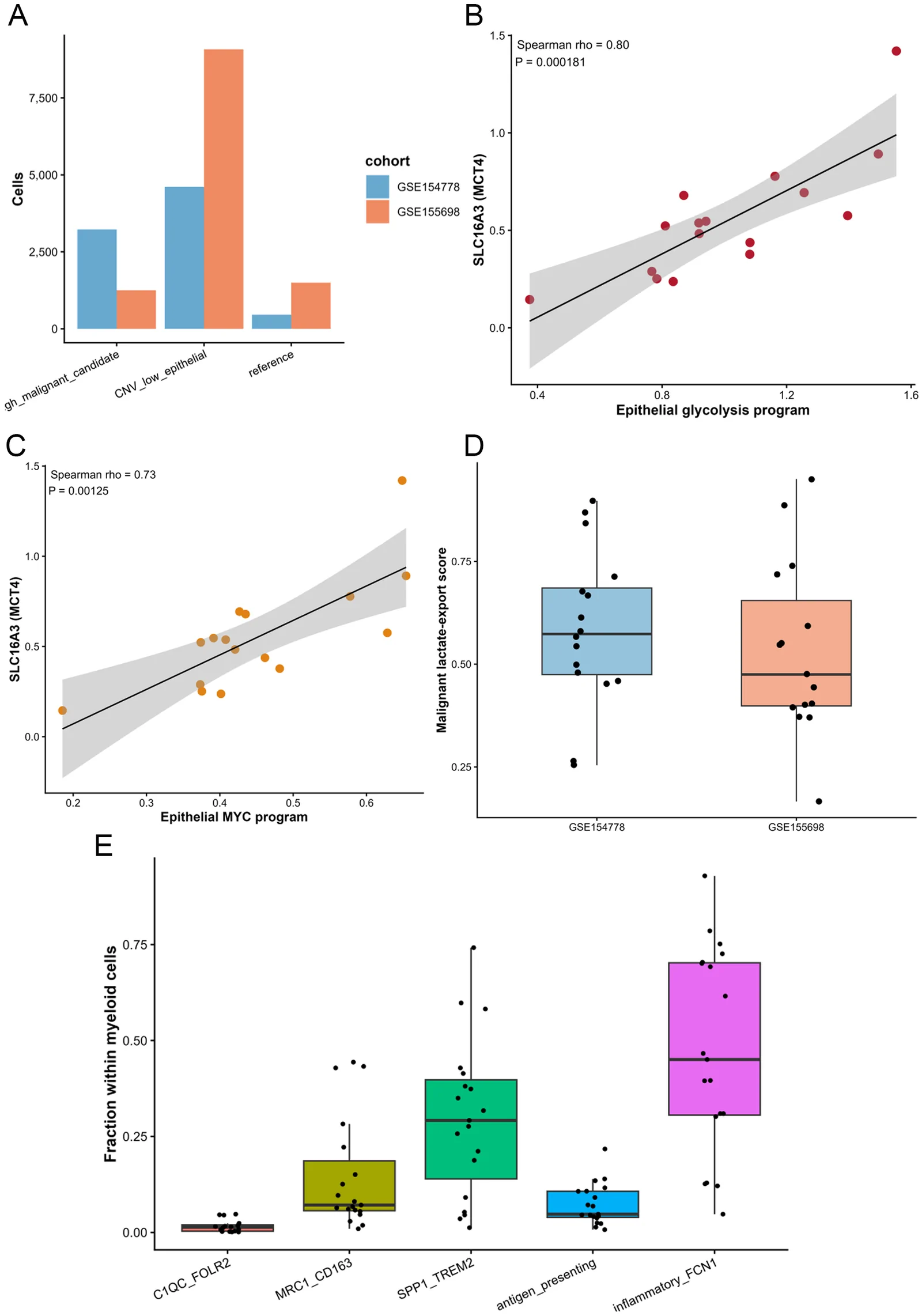
Single-cell characterization of the malignant MYC-MCT4 lactate-export program. **(A)** CNV-window candidate states. **(B, C)** Patient-level glycolysis/MYC relationships with MCT4. **(D)** Lactate-export scores across cohorts. **(E)** Macrophage-state composition.

### 3.3 MYC and LDHA support tumor-intrinsic malignant phenotypes

In the original PANC-1 experimental series, siRNA reduced MYC or LDHA expression and decreased proliferation, clonogenic growth, migration and invasion (**Figures 3A–F**). Immunohistochemical images from the Human Protein Atlas showed tumor-associated expression of both proteins (**Figure 3G**). These findings support tumor-intrinsic roles for MYC and LDHA.

**Figure 3:**
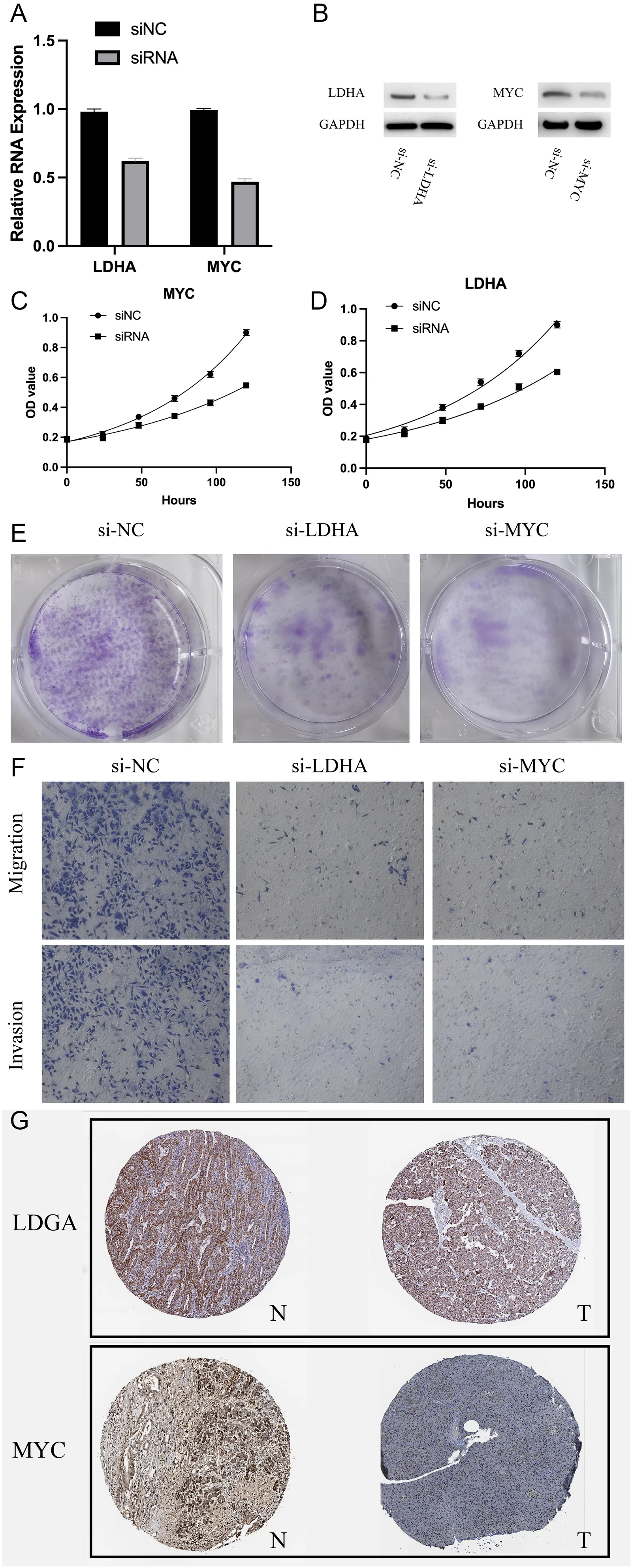
Tumor-intrinsic effects of MYC and LDHA silencing. **(A)** Relative mRNA expression of LDHA and MYC in PANC-1 cells 48 h after siRNA transfection. **(B)** Western blot analysis of LDHA and MYC protein levels 72 h after transfection; GAPDH served as the loading control. **(C, D)** CCK-8 proliferation assays of PANC-1 cells transfected with si-NC or si-MYC (C) or si-LDHA **(D)**. **(E)** Colony formation assays. **(F)** Transwell migration (upper row) and invasion (lower row) assays. **(G)** Representative immunohistochemistry images of LDHA and MYC in normal (N) and pancreatic ductal adenocarcinoma (T) tissue from the Human Protein Atlas. Scale bars and statistical annotations are shown in the images.

### 3.4 MCT4-high malignant regions are associated with a replicated macrophage-receiver pattern

We independently analyzed five GSE274103 and seven GSE235315 spatial samples. Macrophage-receiver scores were higher around MCT4-high malignant cores in 12/12 samples in the innermost ring (median high-core minus low-core difference 0.433; FDR = 0.010) and remained positive across outer rings (**Figure 4**). CAF-receiver effects were heterogeneous, and a CD8-effector exclusion signal was not independently replicated. Subsequent analyses therefore focused on the macrophage-receiver association.

**Figure 4:**
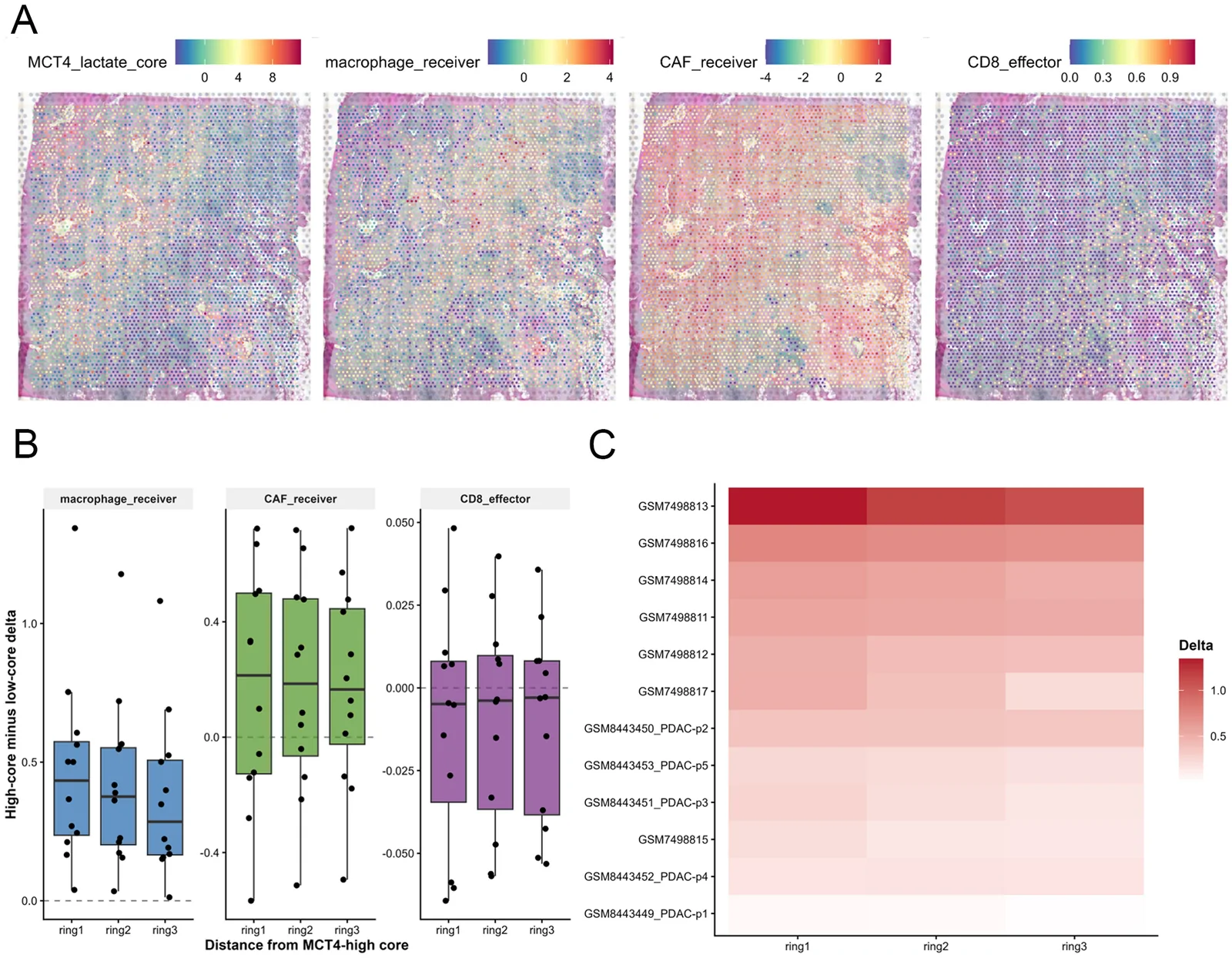
MCT4-associated spatial macrophage-receiver patterns across two cohorts. **(A)** Representative spatial programs. **(B)** Ring-wise high-core minus low-core differences across 12 samples. **(C)** Sample-resolved macrophage receiver effects.

### 3.5 The SPP1/TREM2 program shows the strongest macrophage-state association

State-resolved spatial analysis identified SPP1/TREM2 macrophages as the most consistent receiver population (ring 1: 12/12 positive; median difference 0.613; FDR = 0.006). Inflammatory FCN1 macrophages were also enriched, whereas MRC1/CD163 and C1QC/FOLR2 effects were smaller (**Figure 5A**). SPP1, GPNMB, SLC16A1, LGALS3, VEGFA, CXCL8 and CTSB were repeatedly enriched near MCT4-high cores (**Figure 5B**). Along the FCN1-to-SPP1/TREM2 continuum, GPNMB, APOE, TREM2, SPP1, LDHB and SLC16A1 increased, while S100A8/S100A9 decreased (**Figure 5C**). Patient-aware pseudobulk analysis implicated oxidative phosphorylation, cholesterol homeostasis and MYC-associated programs, consistent with metabolic adaptation rather than only numerical accumulation (**Figure 5D**).

**Figure 5:**
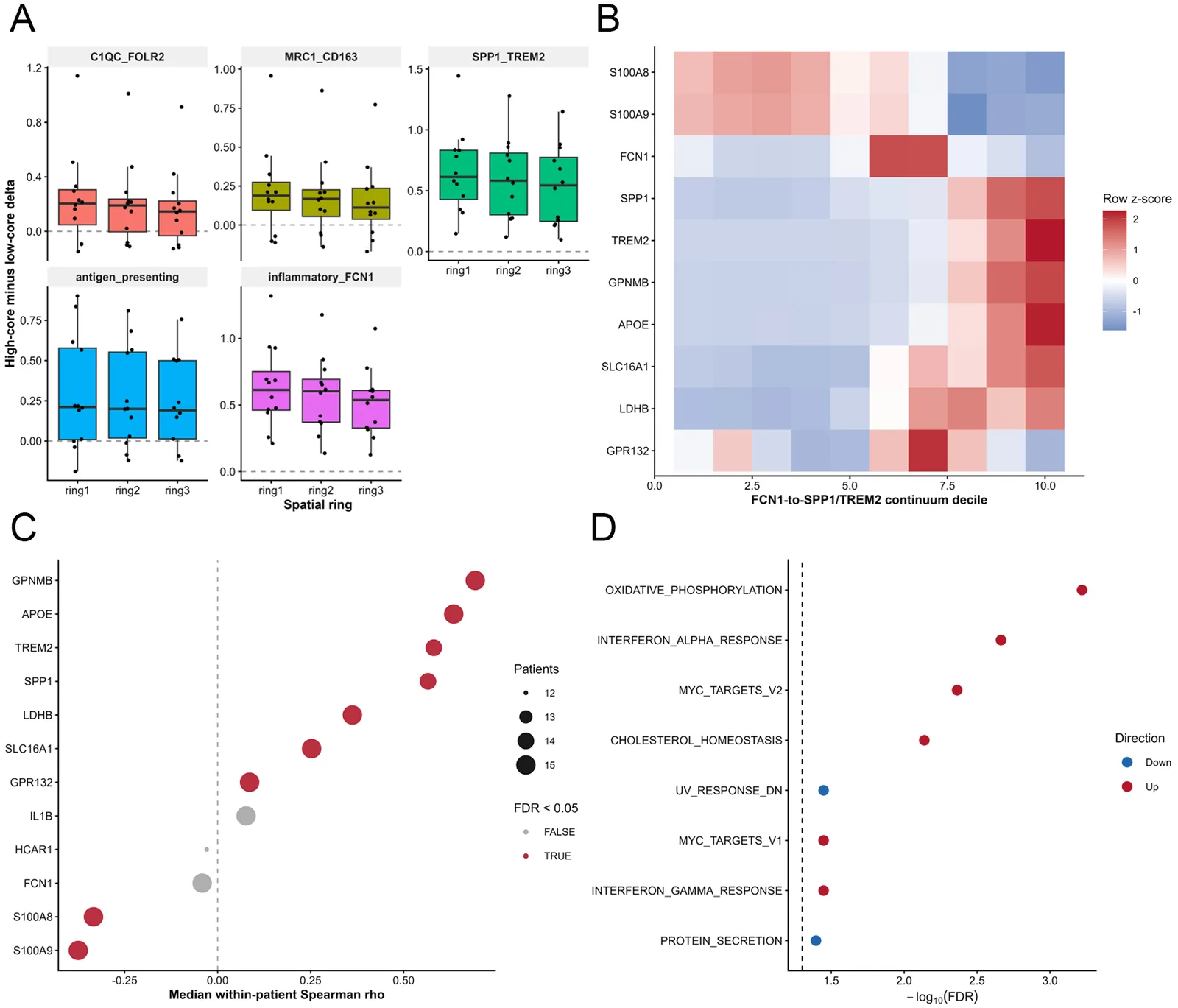
Spatial and transcriptional features of SPP1/TREM2 macrophages. **(A)** Radial enrichment by state. **(B)** State-continuum heat map. **(C)** Patient-level gene trends. **(D)** Pseudobulk pathway enrichment.

### 3.6 Composition-adjusted sensitivity analysis

We next asked whether the macrophage-receiver pattern survives adjustment for local epithelial and myeloid abundance and library size. After controlling for epithelial abundance, myeloid abundance and library size, the ring-1 adjusted SPP1/TREM2 receiver effect remained positive in 10/12 sections. The median section-level high-core minus low-core difference was 0.103. The value 0.017 is the nominal Wilcoxon P value across the 12 section-level differences, not a second effect-size estimate; **Figure 6B** has been interpreted accordingly. After correction over the complete target-by-ring family used in this sensitivity analysis, FDR was 0.071 and therefore statistically non-significant (**Figures 6A–C**). Cohort-stratified analyses were directionally heterogeneous and underpowered (five and seven sections). The adjusted receiver effect remained positive in most sections (**Figure 6A–C**), and **Figure 6D** summarizes the resulting donor-receiver working model. We then turned to functional perturbation to test whether the export axis and the macrophage state are functionally coupled.

**Figure 6:**
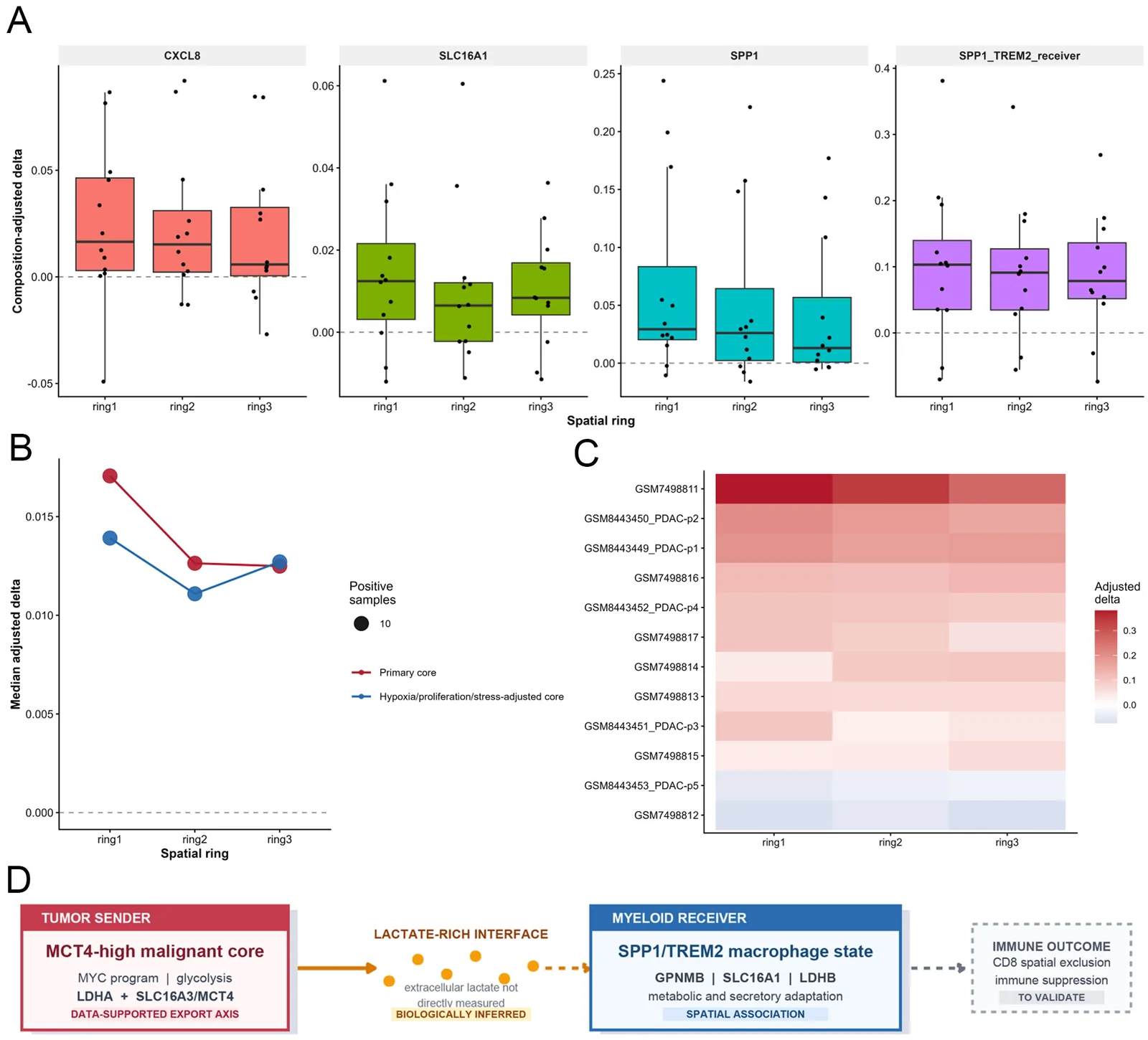
Composition-adjusted sensitivity analyses and proposed working model. **(A–C)** Composition-adjusted section-level sensitivity analyses. Points represent tissue sections; boxes summarize section-level high-core minus low-core differences. The median adjusted receiver difference was 0.103, whereas 0.017 was the nominal Wilcoxon P value; FDR = 0.071 after family-wise Benjamini-Hochberg correction. **(D)** Working model distinguishing measured colocalization from proposed, untested immune consequences.

### 3.7 Tumor lactate-export perturbation modifies conditioned-medium effects *in vitro*

Because MYC, LDHA and SLC16A3 silencing is pleiotropic and can alter glycolytic flux, redox balance, proliferation and multiple conditioned-medium components, the following experiments test whether lactate is one contributing correlate rather than the sole mediator. Silencing MYC, LDHA or SLC16A3 reduced extracellular lactate in PANC-1 and MIA PaCa-2 cells, with comparatively modest effects on glucose and controlled pH (**Figures 7A–D**). The same perturbations reduced tumor-cell viability, while lactate add-back partially rescued the SLC16A3-knockdown phenotype (**Figure 7E**). Conditioned medium from MYC-, LDHA- or SLC16A3-silenced tumor cells reduced macrophage SPP1, TREM2, GPNMB, APOE, SLC16A1/MCT1, LDHB and MRC1 expression; lactate add-back restored part of this response, whereas the pH/osmolarity control did not (**Figures 7F, G**). Macrophage lactate uptake decreased after exposure to knockdown-conditioned medium and increased after lactate add-back (**Figure 7H**). Macrophages exposed to control conditioned medium reduced CD8 proliferation and IFN-γ, while this effect was weaker after MYC, LDHA or SLC16A3 perturbation and partly returned with lactate add-back (**Figures 7I, J**). Lactate add-back rescued these phenotypes only partially, identifying lactate as one contributing mediator alongside other perturbation-induced changes in the conditioned medium.

**Figure 7:**
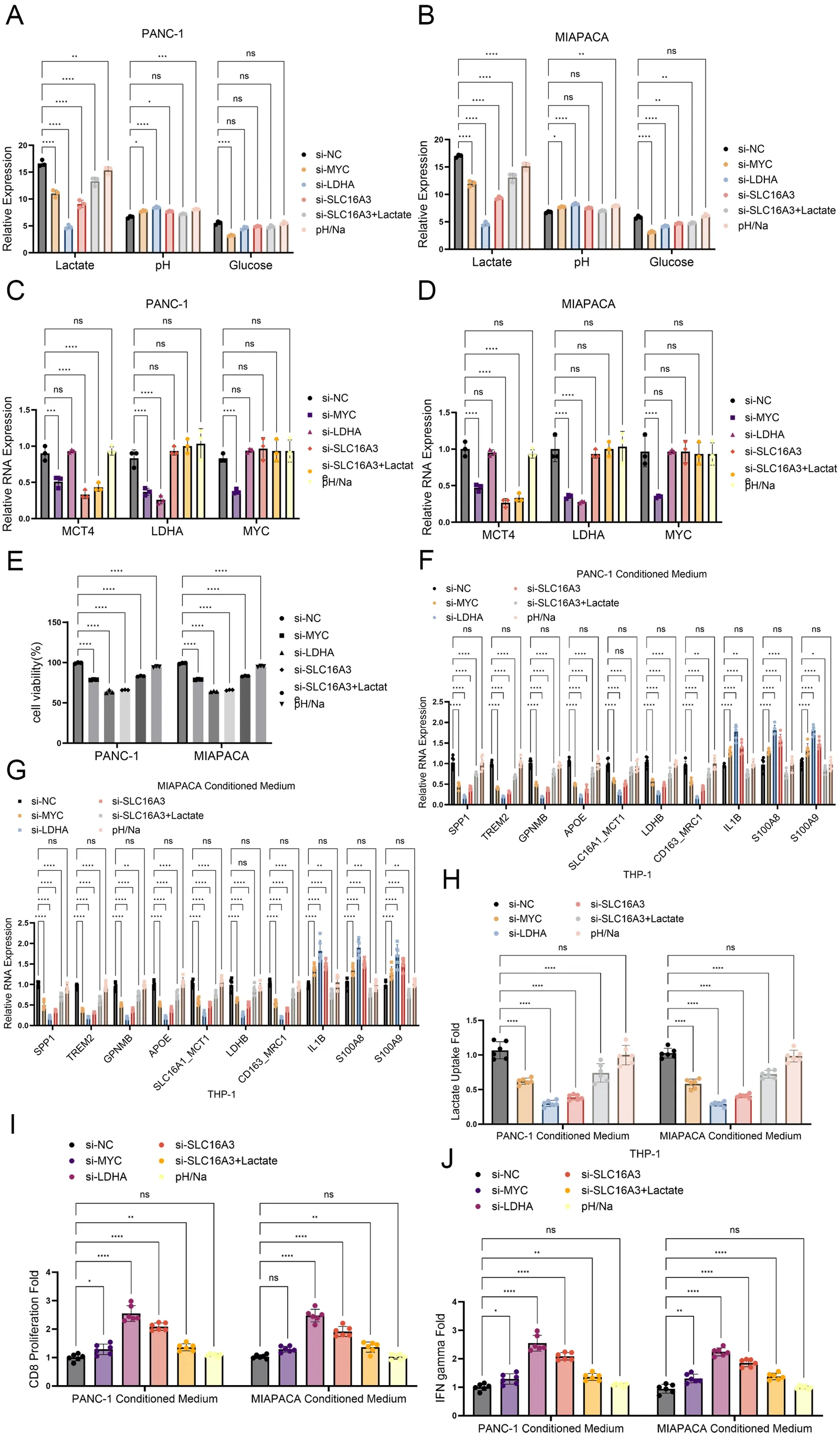
*In vitro* effects of lactate-export perturbation on macrophage and CD8 responses. **(A–D)** Lactate, pH, glucose and target-gene responses after MYC, LDHA or SLC16A3 silencing in PANC-1 and MIA PaCa-2 cells. **(E)** Tumor-cell viability, demonstrating that gene silencing also affected viable-cell abundance. **(F, G)** THP-1 macrophage-state genes after conditioned-medium exposure. **(H)** Lactate uptake. **(I, J)** CD8 proliferation and IFN-γ. Sodium L-lactate was added back to the concentration measured in the corresponding control conditioned medium; pH and osmolarity controls were matched to the add-back condition. Each assay was repeated in three independent experiments; plotted points show the available replicate measurements.

### 3.8 Survival associations vary across independent cohorts

Among 177 TCGA-PAAD patients (93 OS events), the continuous combined score was associated with OS (HR per SD 1.64, 95% CI 1.24-2.17; P = 5.26x10^-4) and remained associated after adjustment (HR 1.55, 95% CI 1.16-2.06; P = 0.00295). GSE62452 included 65 patients and 49 events (HR 1.47, 95% CI 1.04-2.08; P = 0.031). GSE71729 included 123 patients and 83 events and did not reproduce the direction (HR 0.88, 95% CI 0.71-1.09; P = 0.237). All available cases with complete OS time, event status and nine-gene expression were retained; missing covariates were handled by complete-case analysis only in adjusted models. Across all three eligible cohorts, the fixed-effect estimate was HR 1.17 per SD (95% CI 1.00-1.36; P = 0.048), but heterogeneity was substantial (I²=85.9%). The random-effects estimate was non-significant (HR 1.27, 95% CI 0.83-1.94). Leave-one-out estimates were necessarily dominated by which discordant cohort was omitted and did not establish robustness. The non-significant TCGA median-split OS curve is compatible with information loss from dichotomization and possible nonlinearity, but neither explanation was formally tested. The cohort-dependent pattern delineates the clinical boundary of the combined score across the three eligible cohorts (**Figure 8**).

**Figure 8:**
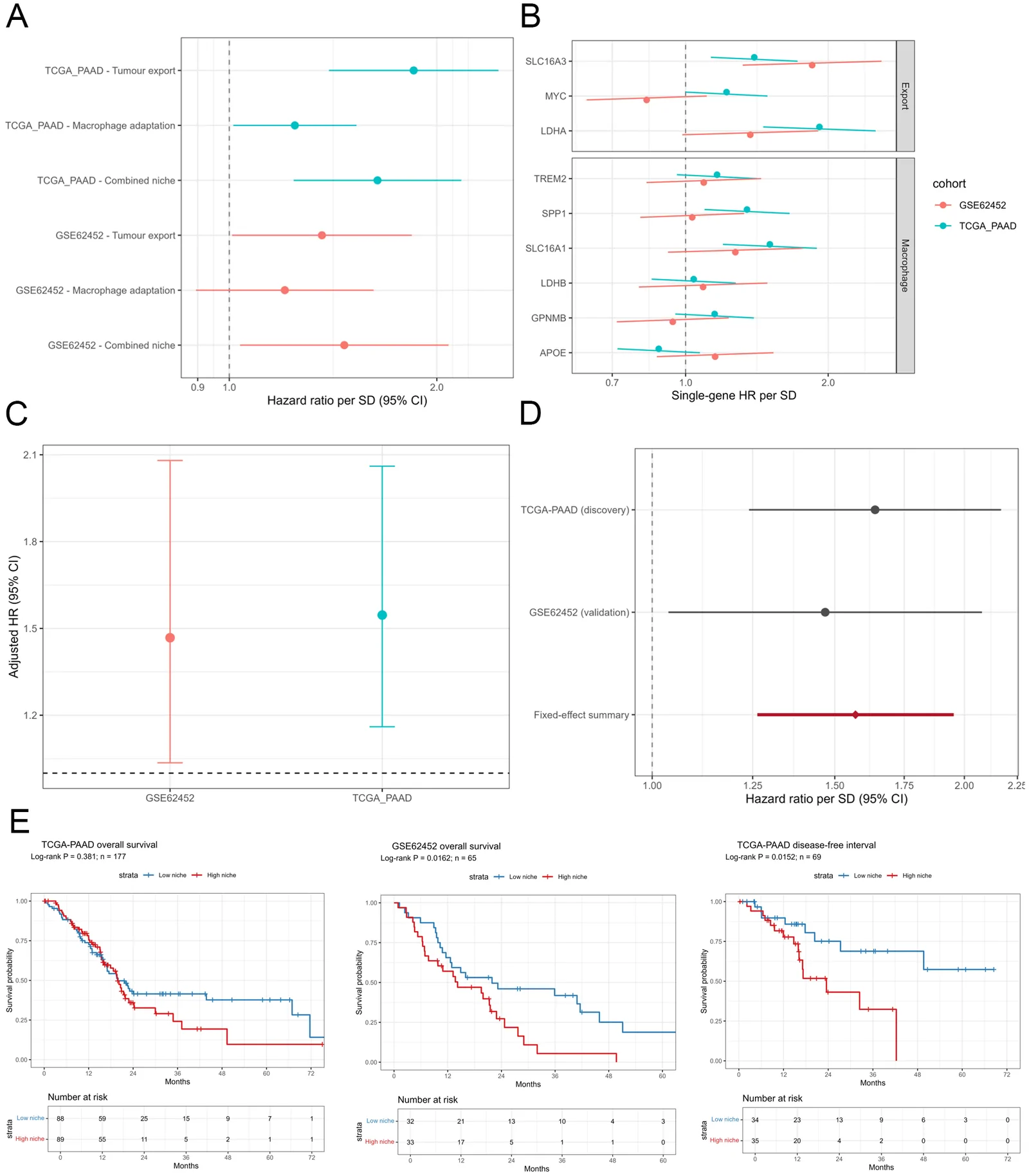
Cohort-dependent survival associations of the combined lactate-export macrophage expression score. **(A)** Exploratory continuous axis and combined-score Cox models for all eligible cohorts. **(B)** Single-gene effects. **(C)** Clinically adjusted estimates where covariates were available. **(D)** Fixed- and random-effects summaries including TCGA-PAAD, GSE62452 and GSE71729; the two-cohort estimate is descriptive only. **(E)** Median-split Kaplan-Meier visualizations shown for descriptive purposes. Hazard ratios are shown with 95% confidence intervals.

### 3.9 Exploratory immune and stromal analyses show weaker or inconsistent associations

Exploratory analyses of dendritic-cell and Treg programs, NETosis/neutrophils, CAF states and Schwann-cell programs identified cohort- or definition-dependent associations but no effect as strong or consistently replicated as the macrophage receiver signal (**Supplementary Figures 6**-**9**). These findings indicate that the spatial association is specific to the SPP1/TREM2 macrophage receiver program rather than a generalized feature of immune or stromal accumulation in PDAC.

## 4 Discussion

Across the datasets examined here, an epithelial MYC-LDHA-MCT4 expression program coincided with an SPP1/TREM2 macrophage program in PDAC. The unadjusted spatial pattern was observed in 12 specimens from two cohorts. After adjustment for epithelial and myeloid abundance and library size, the direction remained positive in 10 specimens, but the analysis did not meet the FDR threshold (FDR = 0.071). Because lactate concentration and flux were not measured in the spatial samples, the data define an expression-based spatial association rather than a demonstrated lactate gradient or a MYC-LDHA-MCT4-driven macrophage niche.

Transcript abundance is not equivalent to metabolite concentration or flux. We use the term expression-based lactate-export program operationally because the score combines genes linked to glycolytic lactate production and monocarboxylate transport, including LDHA and SLC16A3, and because the tumor-cell perturbation experiments show concordant changes in extracellular lactate. This terminology does not imply that a spatial lactate gradient was measured or that the inferred receiver cells imported tumor-derived lactate. Visium spots are multicellular, transporter abundance does not specify net transport direction, and transcript-protein relationships may vary by hypoxia, nutrient availability and post-translational regulation. Accordingly, the spatial results should be interpreted as colocalization of transcriptional programs that generates a testable metabolic hypothesis. Validation will require direct spatial metabolomics, enzymatic or mass-spectrometric lactate mapping, genetically encoded lactate-sensor imaging, or isotope-tracing approaches capable of resolving donor and receiver compartments. Until such data are available, ‘lactate-export’ and ‘niche’ denote transcriptional surrogates rather than measured metabolite transfer.

The phase II CXCR4 study served as clinical motivation rather than mechanistic evidence. CD8 T-cell infiltration increased in most paired samples, whereas no objective responses were recorded and macrophages remained prominent. This observation suggests that recruitment and function can be separable features of antitumor immunity, but the present analyses do not connect CXCR4 or PD-1 signaling directly to the MYC-LDHA-MCT4 axis. Moreover, spatial CD8 exclusion was not consistently replicated. Thus, the trial data frame the clinical question but do not establish that macrophages caused treatment resistance.

SPP1/TREM2 macrophages showed the largest radial difference near MCT4-high regions and expressed GPNMB, SLC16A1, LDHB and lipid- and oxidative-metabolism programs (36). These features are compatible with metabolic adaptation, but neither transporter expression nor pathway enrichment measures lactate uptake. SPP1/TREM2 macrophages are heterogeneous, and TREM2 perturbation can have context-dependent effects in PDAC. Independent multiplex imaging is needed to confirm direct proximity among MCT4-high malignant cells, SPP1/TREM2 macrophages and CD8 T cells at single-cell resolution. Until such validation is available, the spatial findings should be interpreted as spot-level colocalization of expression programs.

Other cellular programs were less consistent. CAF-receiver and CD8-effector results varied between spatial cohorts, while Treg, dendritic-cell, neutrophil/NETosis and Schwann-cell analyses were exploratory. Gao et al. recently integrated single-cell, spatial and GSE62452 data to describe lactate-metabolism heterogeneity and immune associations in PDAC (26). Our incremental contribution is narrower: prioritization of the MYC-LDHA-SLC16A3 axis, section-level proximity to an SPP1/TREM2-centered macrophage state across two Visium cohorts, and conditioned-medium perturbation. The general lactate-PDAC immune relationship is not claimed as novel. Because Visium spots are multicellular and no spatial lactate measurement or permutation null was available, the term niche refers only to a colocalized expression pattern.

The perturbation experiments provide functional context but do not establish a lactate-specific mechanism. MYC and LDHA perturbation broadly affects glycolytic flux, NAD+/NADH balance, anabolic metabolism and proliferation, whereas SLC16A3 perturbation can alter proton-coupled metabolite transport. Reduced lactate is therefore one correlated change among potentially many altered conditioned-medium components. Lactate add-back partly reversed the macrophage and CD8-associated readouts under matched pH/osmolarity conditions, but incomplete rescue leaves other metabolites and secreted factors plausible. THP-1 is a monocytic leukemia cell line; even after differentiation, its epigenetic state and metabolic plasticity differ from those of primary tumor-associated macrophages. Moreover, two-dimensional conditioned-medium assays do not reproduce the three-dimensional stromal barrier, hypoxic and acidic gradients, nutrient competition or multicellular feedback present in PDAC. Thus, the *in vitro* results support only the possibility that lactate contributes under the tested conditions and cannot be directly extrapolated to *in vivo* causality. Confirmation in donor-derived macrophages, selective MCT4 inhibition or rescue, and direct matched-pH lactate exposure would provide stronger specificity.

The survival analysis further illustrates the limits of generalization. Continuous scores were associated with outcome in TCGA-PAAD and GSE62452, but the effect was absent and slightly reversed in GSE71729. GSE71729 was retained in all-cohort fixed- and random-effects summaries rather than excluded. Its two-channel microarray platform, available sample composition and incomplete treatment metadata differ from TCGA RNA sequencing and the GSE62452 platform; tumor cellularity, classical/basal subtype distribution and treatment context may also contribute to directional inconsistency. These explanations remain hypotheses because harmonized covariates were unavailable. Including all three cohorts produced high heterogeneity and a nonsignificant random-effects estimate, while TCGA median-split overall survival was also not significant. The discordant cohort therefore defines an important boundary on portability and precludes presentation of the score as a validated prognostic model.

The relationship between the expression program and PDAC genomic or transcriptomic subtypes remains unresolved. Future studies should test whether the score varies with KRAS allelic state or dosage, TP53/CDKN2A/SMAD4 alterations, and classical versus basal-like tumor programs. A mechanistic *in vivo* test could combine tumor-cell-selective Mct4 deletion or pharmacological MCT4 inhibition with anti-PD-1 treatment in an immunocompetent KPC-derived model. Serial assessment of extracellular lactate, SPP1/TREM2 macrophage states, CD8 infiltration and function, and tumor control would determine whether disrupting transport changes the proposed spatial relationship and improves checkpoint response.

Several limitations remain unresolved. Public datasets differed in sampling, treatment exposure and platform. Cell-flow reporting could not reconstruct every prefiltering intermediate from processed public matrices, mapping uncertainty was not used to exclude spots, and expression-based CNV and receiver scores may retain composition bias. The spatial analysis lacked continuous-distance modeling, within-section permutation, overlap correction and explicit edge correction. The adjusted spatial family was statistically non-significant (FDR = 0.071). Survival models lacked full proportional-hazards, nonlinearity and influence diagnostics and showed marked cross-cohort heterogeneity. No independent tissue cohort was examined by multiplex immunofluorescence, and functional studies did not include primary macrophages, selective transport intervention or an immune-competent model. These limitations prevent causal, spatial-transfer and clinical-prediction claims.

In summary, the study identifies a repeated spatial and transcriptional relationship between an epithelial MYC-LDHA-MCT4 program and an SPP1/TREM2 macrophage state in PDAC. *In vitro* perturbation and partial add-back results are compatible with, but do not prove, a contribution from lactate. The resulting model is a testable framework for tumor-myeloid metabolic interaction rather than a demonstrated causal pathway or a validated prognostic tool.

## Data Availability

The original contributions presented in the study are included in the article/**Supplementary Material**. Further inquiries can be directed to the corresponding author/s.

## Supplementary material

Supplementary Figure 1 Original clinicopathological expression analyses.

Supplementary Figure 2 Original overall- and disease-free-survival analyses.

Supplementary Figure 3 Original signaling and proliferation analyses, restored in the original layout.

Supplementary Figure 4 Single-cell quality control, annotation and marker validation.

Supplementary Figure 5 Macrophage subclustering and independent tumor-versus-blood validation.

Supplementary Figure 6 Exploratory dendritic-cell and regulatory-T-cell analyses.

Supplementary Figure 7 Exploratory neutrophil and NETosis-related analyses.

Supplementary Figure 8 Exploratory CAF and Schwann-cell analyses.

Supplementary Figure 9 Macrophage-state continuum, sensitivity and candidate-interaction analyses.

Supplementary Figure 10 Spatial maps for all 12 samples across two independent cohorts.

Supplementary Table 1 Dataset eligibility, sample flow and endpoint availability.

Supplementary Table 2 Complete gene sets used for principal and exploratory expression scores.

Supplementary Table 3 Analysis-to-test reporting matrix.
