# Supplementary material for: A MYC-LDHA-MCT4 expression program marks an SPP1/TREM2-centered macrophage state in pancreatic ductal adenocarcinoma
Source: Front Immunol. 2026 Oct 5;17:1978427. doi: 10.3389/fimmu.2026.1978427 (PMC13677877; doi:10.3389/fimmu.2026.1978427)
Supplement: Supplementary file 1 [file Table1.docx]

# Supplementary tables

Supplementary Table 1. Dataset eligibility, sample flow and endpoint availability.

| **Dataset** | **Modality** | **Starting material** | **Analyzed** | **Events** | **Role** | **Missing/exclusions** |
| --- | --- | --- | --- | --- | --- | --- |
| NCT04177810 IMC | IMC | 21 treated patients | 11 paired tissues | 0 objective responses | Clinical motivation | Unpaired specimens excluded from paired tests |
| GSE154778 | scRNA-seq | 14,926 matrix cells; 16 patients | 14,102 singlets; 9,722 epithelial; 527 immune | NA | Discovery scRNA | 824 cells removed by QC/doublet filtering |
| GSE155698 | scRNA-seq | Processed sample matrices | 50,086 post-QC tissue cells | NA | Independent scRNA/reference | Prefilter counts unavailable from processed input |
| GSE217845 | scRNA-seq | 6 tumor-blood patient pairs | 107,868 post-QC cells | NA | Myeloid validation | Prefilter counts unavailable from processed input |
| GSE274103 | Visium | 5 treatment-naive sections | 5 sections | NA | Spatial cohort 1 | Tissue spots with valid coordinates/scores |
| GSE235315 | Visium | 7 sections | 7 sections | NA | Spatial cohort 2 | Tissue spots with valid coordinates/scores |
| TCGA-PAAD | Bulk survival | 177 eligible | 177 OS; 175 adjusted | 93 OS | Discovery survival | Adjusted model used complete covariates |
| GSE62452 | Bulk survival | 65 eligible | 65 | 49 OS | External sensitivity | Complete OS and nine genes required |
| GSE71729 | Bulk survival | 123 eligible | 123 | 83 OS | External sensitivity | Retained despite discordant direction |

Supplementary Table 2. Complete gene sets used for principal and exploratory expression scores.

| **Program** | **Genes** | **Use** |
| --- | --- | --- |
| Epithelial | EPCAM, KRT8, KRT18, KRT19, KRT7, MUC1 | Core/composition |
| Lactate production | SLC2A1, HK2, PFKP, ALDOA, GAPDH, PKM, LDHA | Spatial core |
| Lactate export | SLC16A3, LDHA, CA9, HIF1A, SLC2A1, SLC9A1 | scRNA; spatial core |
| Myeloid | LST1, TYROBP, FCER1G, AIF1, CTSS, LYZ | Composition adjustment |
| SPP1/TREM2 state | SPP1, TREM2, GPNMB, APOE, LPL, CTSB | Principal receiver |
| Lactate reception | SLC16A1, LDHB, GPR132, HCAR1 | Principal receiver |
| CD8 effector | CD3D, CD8A, CD8B, CCL5, NKG7, PRF1, GZMB, GZMH | Spatial secondary |
| Treg | FOXP3, IL2RA, CTLA4, TIGIT, IKZF2, TNFRSF4 | Exploratory |
| cDC1 | CLEC9A, XCR1, BATF3, CADM1, IRF8 | Exploratory |
| cDC2 | CD1C, FCER1A, CLEC10A, CD1E | Exploratory |
| LAMP3 DC | LAMP3, CCR7, FSCN1, CCL19, CCL22 | Exploratory |
| pDC | CLEC4C, GZMB, TCF4, IL3RA, IRF7 | Exploratory |
| NET machinery | PADI4, MPO, ELANE, CYBB, NCF1, NCF2 | Exploratory |
| myCAF | ACTA2, TAGLN, MYL9, TPM2, CNN1, COL1A1 | Exploratory |
| iCAF | IL6, CXCL12, CFD, C7, COL14A1, DPT | Exploratory |
| apCAF | HLA-DRA, HLA-DPA1, HLA-DPB1, CD74, CIITA | Exploratory |
| Schwann | S100B, SOX10, PLP1, MPZ, PMP22, SLC1A3 | Exploratory |
| Hypoxia | CA9, ADM, BNIP3, NDRG1, HILPDA, VEGFA | Sensitivity |
| Proliferation | MKI67, PCNA, MCM2, MCM4, TOP2A, TYMS | Sensitivity |
| Survival export | MYC, LDHA, SLC16A3 | Exploratory survival |
| Survival macrophage | SPP1, TREM2, GPNMB, SLC16A1, LDHB, APOE | Exploratory survival |

Supplementary Table 3. Analysis-to-test reporting matrix.

| **Figure** | **Endpoint** | **Unit/n** | **Test/model** | **Multiplicity** | **Effect measure** | **Replicates/error bars** |
| --- | --- | --- | --- | --- | --- | --- |
| 1 | Paired CD8/macrophage fractions | Patient; n=11 | Paired Wilcoxon | BH within IMC family | Median paired change | Individual patients; boxes show distribution |
| 1 | Tumor-blood macrophage states | Patient pair; n=6 | Paired Wilcoxon | BH within state family | Paired difference | Individual patients |
| 1,3 | Program correlations | Patient; n reported per panel | Spearman | BH within family where shown | rho | Patients; fit line descriptive |
| 2 | Tumor-cell phenotypes | Independent experiment; >=3 | ANOVA/post-hoc or Kruskal-Wallis | Post-hoc adjustment | Group difference | Biological repeats; define bars in source panel |
| 4-6 | Radial receiver differences | Section; n=12 (5+7) | Signed-rank | BH over target x ring family | Median high-low delta | Individual sections; boxplots |
| 5 | Macrophage pseudobulk | Patient-by-state | edgeR + ranked enrichment | BH | log fold change/NES | Patient-level pseudobulk |
| 7 | Lactate, genes, CD8 function | Independent experiment; >=3 | ANOVA/post-hoc or Kruskal-Wallis | Post-hoc adjustment | Group difference | Biological repeats; technical wells not treated as n |
| 8 | Overall survival | Patient; 177/65/123 | Cox per SD; meta-analysis | BH for score family | HR with 95% CI | Patients; KM bands where shown |
| S6-S9 | Exploratory immune/stromal scores | Patient or section | Spearman or signed-rank | BH within each analysis family | rho or median delta | Biological units shown |
